# Notes and News

**Published:** 1890-08-02

**Authors:** 


### Notes and News.

Collected and Communicated.

The first annual report of the Clapham Maternity Hospital comes out in neat and handy form, and from the simplicity of its accounts reflects credit on the secretary. The income last year was £457, and there is a balance of £15 at the bankers. The in-patients last year were 64, the maternity out-patients 318, dispensary attendances 8,964. The dispensary is in Stockwell Road, away from the hospital, to diminish the risk of infection.

It seems that a special commissioner of the *Lancet* has been making inquiries concerning the duties, pay, diet, and recreation of hospital nurses, much on the lines followed by the present Select Committee. He says : "What strikes me as being the most urgent demand is for a shortening of the hours of unrelieved work. The twelve-hours system is a cruel strain on a woman's strength and nerve. From one of my reports I learn that three days in every week are thirteen hours and a-quarter long, from 8.30 a.m. to 9.45 p.m. To such toilers recreation time must come too late to find in them the capacity for enjoyment." If this inquiry were fully carried out, the facts deduced would be very valuable; but it is necessary that the truth of each answer should be tested.

The secretary of the Hospital for Epilepsy and Paralysis sends us the following statement : Debt 10 years ago, £2,000 ; debt on November 30th, 1882 (when present secretary took office), £1,548. At the end of June, 1890, the available balance, after allowing for all liabilities, was £1,000, including £500 invested. Also through the munificence of an anonymous donor, who gave £1,200 for this purpose, the committee have purchased the lease (10 years unexpired), and have bought an annuity to provide for the payment of the ground rent—£150 a-year for 10 years is thus saved to the hospital, which is now rent free for that period. The same anonymous benefactor has promised £2,000 towards a building and furnishing fund, which the committee will proceed to raise—£20,000 being required for this purpose. If ten more donors would follow example, the fund would be raised without any expenditure on appeals.

The National Hospital for the Paralysed and Epileptic contains 175 beds and cots. Great pressure has been exercised on this hospital lately, and the committee plead for £1,200 to repay a loan they had to contract this year. There were 647 in-patients last year, and the attendances of the out-patients were 25,115. There is a pension fund for incurable cases. The report is very full and clear, and opens with a touching extract from the speech of the Archbishop of Canterbury, at the festival in May, 1889.

On Saturday last Viscountess Wolseley opened a new cottage hospital at Shooter's Hill, near Woolwich. Her ladyship drove over from the Ranger's House, Greenwich Park, the residence of Lord Wolseley, and was received at the hospital by General Williams. The Rev. S. G. Scott, Rector of Woolwich, in addressing Lady Wolseley, said that in the jubilee year of Her Majesty, Woolwich desired to erect some permanent expression of their loyalty to the throne, which should at the same time be a boon to the neighbourhood. The population numbered 90,000, and consisted chiefly of the artizan class, who were a considerable distance from the London hospitals, therefore the present building was erected. The hospital was designed by Mr. J. O. Cook, and built by Messrs. Coombs and White, the funds being chiefly collected through the perseverance of Mr. W. Woodford, backed up by the friendly societies of the district.

The annual report of Louth Hospital and Dispensary is out. For the year ending May 31st last, 85 patients have been treated in the Hospital. In the same period 757 cases were treated in the Dispensary, making a total of in and out patients during the 12 months, of 842. The attending surgeon made 2,491 attendances in the dispensary, and 264 visits to patients at their own homes. The income of the institution for the past year amounted to £680 10s. 10d., being £65 14s. 3d. less than the previous year. This difference is mainly attributable to the fact that the concert did not realise half as much as the special effort made last year in connection with the Hospital Saturday Fund. The expenditure amounted to £704 8s. 2d., thus leaving a balance due to the treasurer of £4 0s. 6d. The Endowment Fund is the same now as it was last year, and amounts to £6,700. There is a Samaritan Fund in connection with the hospital. The medical staff, matron, and nurses are thanked for loyal services.

American women with commendable 'cuteness have taken advantage of the poverty of the John Hopkins Hospital to make a bargain with the authorities which should be to the benefit of all. The somewhat straitened financial condition of that institution suggested to many prominent women of the country the possibility of contributing to its relief and at the same time hastening the establishment of a medical school in which the benefits of the highest medical training shall be afforded. To this end they propose to raise the sum of $200,000, which is to be given to the trustees on the distinct condition that women whose previous training has been equivalent to that of an ordinary preliminary medical course shall be admitted to the institution on precisely the same terms as men. Committees are at work in six of the principal cities, including Boston and San Francisco, and considerable contributions have already been made to the furtherance of the movement. It is not designed that this medical school shall take the place of the ordinary schools for women now in existence, but that it shall supply opportunities for that advanced medical training which they are now obliged to seek in foreign schools. The prominence of the ladies engaged in this enterprise, together with the obvious desirableness of the school proposed, leave no room for doubt that it will be successful.

The following vacancies are announced this week, the amount of the salary in each case being given after the name of the institution :—

**Resident Medical Officers.**

Kent County Asylum, fourth Assistant Medical Officer—£150, rooms, etc.

Manchester Royal Eye Hospital, House Surgeon—£70, board, etc.

Cheadle Convalescent Hospital, Resident Medical Officer—£150, board, etc.

Dudley Dispensary, Resident Medical Officer—£130, rooms, etc.

North Staffordshire Infirmary, Assistant House Surgeon.

**Non-Resident Medical Officers.**

Gloucester Friendly Societies, Assistant Medical Officer—£120.

Chelsea Dispensary, Visiting Surgeon.

Barothery Union, Medical Officer—£145.

Carlow Union, Medical Officer—£105.

Counties of Caithness and Sutherland, Medical Officer—£400.

Hebburn Local Board, Medical Officer of Health—£75.

---

The conference on the education of the blind, held at the Royal Normal College, has come to an end, as usual in such cases, without any definite result, but with perhaps an added interest in the cause. Mr. Mundella, M.P., said it was now generally agreed that for the sighted primary education should be compulsory, and, he would add, free. Surely, then, the State should insist on the blind being put on an equality with their more fortunate companions in this respect. The Duchess of Westminster distributed the prizes to the successful pupils of the college.

---

The report of Dr. T. O. Dudfield, medical officer of health for the Kensington district, states that 290 births, viz., 153 males and 137 females, and 217 deaths, 122 being male and 95 females, were registered in the four weeks ending July 12th. The deaths were equivalent to an annual rate of 15·7 per 1,000 persons living, or 1·7 per 1,000 above the decennial average of 14·0. One hundred and seventeen of the deaths were of children under five years of age, including 62 under one year. Forty-two persons died at 60 and upwards. The deaths from the principal diseases of the zymotic class were 67 (or 31 above the corrected decennial average), viz., measles 38, scarlet fever 1, diphtheria 5, whooping-cough, 8, enteric fever 1, and diarrhœa 14. Thirty-one deaths were caused by the diseases of the respiratory organs, including bronchitis 14, and pneumonia 8. The deaths from phthisis were 12, from diseases of the heart 8, and from tubercular or scrofulous diseases of children under five years of age 8.

---

The Prince and Princess of Wales received a very warm welcome in Southwark last week, and, as usual, won all hearts by their gracious sympathy with the sick folk they visited. Three public engagements were fulfilled by the hard-worked Heir Apparent to the Throne in one afternoon ; first a visit to the Evelina Hospital, then laying the foundation-stone of a church, and then the foundation-stone of the New South London Ophthalmic Hospital. At the Evelina the small patients were cheerily chatted to by their Royal visitors, and Princess Victoria paid great attention to two very tiny children ; all the party were pleased with what they saw of the hospital. At the Royal South London Ophthalmic, the design of the new building was explained to the Prince and Princess. The architect (Mr. Keith D. Young) was mentioned as the pioneer in England of modern scientifically-dictated hospital construction, as exemplified in the London Fever Hospital, in the circular-ward system, and in other hospitals. The Prince of Wales, in accepting the address, assured the Governors that the work in which they were engaged was most gratifying, and he and the Princess were most pleased to assist in so useful an undertaking. The Princess allowed one of the wards in the new building to be named after her. The decorations in the neighbourhood of the hospital were brilliant and extensive.

Dr. Lagneau, of Paris, proposes to levy a tax on bachelors to be used to support the foundling hospitals. There is a fine irony in this idea, and in other twelve plans for the increase of population which Dr. Lagneau has solemnly put before the Government. If only Dr. Lagneau could be a reporter for a society paper during the month of July in London, he would get a disgust of marriages, which are now being reeled off at the rate of eight or nine a day. The doctor must surely be a much married man himself since he considers that nothing short of a heavy tax on the happy bachelors can make things fair.

---

Barberton Hospital Board met on May 2nd, and the Cape mail has just brought us a full report of the proceedings. During last year the patients admitted were 193 white, and 62 natives, against 152 white and 56 natives treated the year before. The rush on the hospital has been so great that some of the urgent cases have had to be treated at hotels and private houses. Malarial fever was diagnosed in 109 cases, and there were eight deaths from it. There were eight cases of acute alcoholism, and three deaths. The cost per patient per month was £5 11s. 11d. The nursing staff are recruited from Kimberley Hospital, and all seems managed in an excellent manner.

---

The annual meeting of supporters of Huddersfield Infirmary was held on July 24th, Mr. Mallinson presiding. The average daily number of patients in the infirmary has been 74; and the average time during which each patient remained under treatment has been 28 days. The number of deaths in the infirmary within 48 hours after admission has been 17. These were cases of severe injury of a hopeless character, and they unduly raise the rate of mortality in the hospital. The percentage of deaths to the whole of the ordinary medical and surgical cases treated has been five ; deducting the deaths that occurred within 48 hours after admission, the percentage is reduced to 3·2. The number of out-patients who have sought relief has been 4,349 as compared with 4,154 last year. The number of home patients visited by the house surgeons was 937, an increase of 48 on the preceding year, and 1,962 cases of minor injuries. The in-patients number 964 against 914 last year, making a total number of patients treated 8,212. Financially, the institution is in a satisfactory position, there being a balance at the bank of £152 19s. 10d. The report of the Meltham Mills Convalescent Home was also satisfactory.

---

Mrs. Griffith Llewellyn has formally opened the new Eye Hospital at Swansea. The building is in the grounds of the General Hospital, and is at some future time to be connected with same by means of a covered way. It is built of native stone, the style being somewhat of the same character as the present General Hospital. The entrance porch is of Bath stone, with carved panels, surmounted with a bold cornice and balusters. It provides, on the ground floor, a large entrance hall, which is also used for a waiting-room for outdoor patients, with a handsome staircase, and a large window ; lighting hall and landing above ; head nurse's room, consulting-room, operating-room, with dark-room, a large ward, 30 ft. by 20 ft., with lavatories, bath-room, and conveniences. These are, throughout the building, fitted up with the latest and most approved system as regards sanitary appliances, the walls being lined with glazed tiles. There is also a kitchen and scullery, with lifts to first floor. The first floor contains a large ward, 30 ft. by 20 ft., smaller ward, three nurses' rooms, with all the usual lavatories, conveniences, etc. The building has been carried out from the designs and under the superintendence of J. Buckley Wilson, F.R.I.B.A., and Glendinning Moxham, M.S.A., architects of the town.

